# Factors influencing participation in colorectal cancer screening—a qualitative study in an ethnic and socio‐economically diverse inner city population

**DOI:** 10.1111/hex.12489

**Published:** 2016-08-22

**Authors:** Nimarta Dharni, David Armstrong, Guy Chung‐Faye, Alison J. Wright

**Affiliations:** ^1^ Bradford Institute for Health Research Bradford Teaching Hospitals NHS Foundation Trust Bradford UK; ^2^ Department of Primary Care and Public Health Services King's College London London UK; ^3^ King's College Hospital NHS Foundation Trust London UK

**Keywords:** bowel cancer screening, colorectal cancer screening, ethnicity, FOBt, psychological beliefs, socio‐economic status

## Abstract

**Background:**

Ethnic and socio‐economic inequalities have been reported in the uptake of colorectal cancer (CRC) screening. This study aimed to explore the factors affecting CRC screening participation in an ethnically and socio‐economically diverse inner city population.

**Methods:**

Semi‐structured interviews were undertaken with 50 people aged 55–74 years, recruited from GP practices in south‐east London. Participants were from Black African (n=13), Black Caribbean (n=15), White British (n=17), Black other (n=2) and White other (n=3) backgrounds. Participants' socio‐economic status (SES) was assessed using a combined measure of educational attainment, housing tenure and car ownership. Participants' SES varied although there were more participants from less deprived backgrounds than those from more deprived backgrounds. The interview topic guide was informed by the Theoretical Domains Framework. Interviews were recorded, transcribed and analysed using framework analysis.

**Findings:**

Lack of awareness of CRC screening was a barrier for all participants. There were also some notable group differences by ethnicity and SES. Cancer fear was a barrier for White British participants of varying SES. Misunderstanding instructions for completing the guaiac faecal occult blood test (gFOBt) was a barrier for people of low SES regardless of ethnicity. For Black African and Black Caribbean participants, of any SES, religious faith and a perceived civic duty to participate in screening encouraged participation.

**Discussion and conclusions:**

This is the first study to provide detailed information on the separate views of Black African and Black Caribbean participants about screening. Consideration of ethnicity and SES together also allowed us to identify pertinent barriers for particular groups that can be targeted to improve access to screening for those who wish to take part.

## 1 Introduction

The UK national screening programme for CRC was established in 2006 to enable the early detection of CRC in males and females aged between 60 and 74 years. Free at the point of use, screening is offered biennially through a guaiac faecal occult blood test (gFOBt) to detect hidden traces of blood in the faeces, which can be a common occurrence in people with CRC and those with precancerous polyps.^1^ A Cochrane review of gFOBt randomized control trials (RCTs) concluded there was sufficient evidence to suggest that screening can reduce mortality from CRC by 16%.^2^ In England, the national CRC screening programme is delivered independently of primary care. CRC screening also differs from the pre‐existing breast and cervical cancer screening programmes in that it is the first UK mass screening programme to include both men and women and gFOBt completion is undertaken by individuals themselves.

Uptake of many forms of screening varies according to the individuals' socio‐economic status (SES) or ethnicity and colorectal cancer screening is no different. Differences in CRC screening uptake by SES exist even in countries, like the UK, with universal health‐care coverage and screening free at point of use. Therefore, SES disparities in uptake are not simply a result of inability to pay. Instead, SES may affect screening uptake by many pathways, which are comprehensively discussed by von Wagner and et al.^3^ One potential pathway is that people with lower SES experience more frequent life stressors but have fewer resources to cope with them. This may lead to greater pessimism about the future, including expecting screening to be unpleasant and the consequences of being diagnosed with cancer as worse. Greater immediate stressors could also lead to lower concern for future consequences of current health actions, due to the need to cope with present challenges. Undergoing CRC screening involves trading off immediate discomfort and potential embarrassment against the potential future benefits of detecting cancer at an earlier stage. In a trial of screening via flexible sigmoidoscopy, lower SES was related to lower concern for future consequences, which were in turn associated with perceiving fewer benefits and greater barriers to screening, leading to reduced screening uptake.^4^ The incidence of cancer is greater in more socio‐economically deprived individuals, and people with lower SES are often diagnosed at a later stage.^5^ Therefore, individuals with low SES are more likely to have a vicarious experience of cancer with poor prognosis, leading to greater fatalism which deters motivation for screening.

There are also a range of ways in which ethnicity might influence screening uptake. Language barriers may impact ability to understand screening invitations and accompanying information. Different ethnic groups may have different beliefs about health, illness and prevention. A recent systematic review of qualitative studies exploring factors influencing CRC screening uptake^6^ suggested that members of some minority ethnic groups were deterred from screening because they did not see screening as part of their culture, or they felt protected by consuming foods typical of their culture or by using traditional natural remedies. In the UK, CRC screening uptake is lower for individuals from South Asian backgrounds than for White British individuals.^7^ People of South Asian ethnicity may be particularly fatalistic about CRC,^8^ which may be one explanation for this lower uptake. There is a lack of data on CRC screening uptake rates amongst members of other ethnic groups, such as Black African and Black Caribbean individuals. Uptake of CRC screening has been found to be particularly low in south‐east London (approximately 40%;^9^) which probably reflects its high ethnic minority population and levels of socio‐economic deprivation. In the inner city boroughs of Lambeth and Southwark, approximately 26% of residents are from Black/African/Caribbean backgrounds and the areas are amongst the most deprived boroughs in London.^10^

Previous studies of CRC screening uptake have identified a wide range of influences, such as social support, embarrassment, fear, fatalistic beliefs and lack of awareness of CRC as a disease and the role of screening in mitigating the impact of CRC.^8^, ^11^, ^12^, ^13^ However, it is unclear to what extent these “personal” factors map onto the more structural determinants of screening uptake, such as ethnicity and SES. Furthermore, the choice of factors studied has not always been well informed by psychological theory with many studies omitting potentially key determinants of behaviour.

A recurring concern about research examining beliefs about CRC screening in relation to either ethnicity or SES is the lack of consideration of the other factor, making it difficult to establish whether knowledge differences attributed to ethnicity, for example, are due to ethnic differences or variation in SES such as educational attainment. There are also potential limitations of how researchers categorize certain ethnic groups through umbrella terms, such as Afro‐Caribbean, thus overlooking key differences in cultural identity, migration history and language that may contribute to different beliefs about CRC screening.

The Theoretical Domains Framework (TDF; 15) was used to provide a firm theoretical basis to the study, in line with the MRC's guidance on developing and evaluating complex interventions.^14^ A systematic review found that just under half of the qualitative studies of factors affecting CRC screening uptake used a theoretical model, with the most common being the Health Belief Model.^6^ The TDF encompasses a comprehensive range of constructs from numerous psychological theories of the determinants of behaviour change. It was developed and refined through a series of expert consensus exercises.^15^ Although originally developed to understand and change the organizational behaviour of health‐care professionals,^16^, ^17^ more recently the TDF has been used to understand behaviour change in a range of health settings such as uptake of NHS health checks.^18^ The developers of the framework also devised a series of questions to allow the exploration of each domain in relation to different behaviours.^15^ At the time this study was designed, the TDF covered twelve domains and has since been refined to include fourteen domains.^19^ Table S1 shows the TDF domains and their definitions at the time this study was conducted, along with example questions used to probe each domain.

Using the TDF, the present study aimed to explore how the personal beliefs of individuals living in south‐east London map onto individual factors such as ethnicity and SES. By identifying the factors that appear to drive uptake (and non‐uptake) of screening in different groups, strategies that target these underlying processes can be developed to enhance the informed uptake of CRC screening.

## 2 Methods

Ethical approval to conduct this study was received from the NHS Outer North East London Research Ethics Committee (REC reference: 10/H0701/2). Written informed consent was obtained for each participant.

### 2.1 Study population

Recruitment took place at three general practices (one in Lambeth and two in Southwark) from May to September 2010. Practices were identified through academic GP colleagues who suggested suitable practices that were likely to have a large percentage of patients from ethnic minority groups. Participant sampling was purposive to ensure a representative inclusion of males and females aged 55–75 years and those from Black African, Black Caribbean and White British backgrounds. The inclusion criteria included people slightly younger and older than the age range of the UK CRC programme at the time of the study to examine the extent to which views on screening were influenced by age cohorts. Participants with a current cancer diagnosis were not included in the study in recognition of the treatment and personal demands they may have been facing at the time. Moreover, those with a hereditary CRC risk syndrome such as familial adenomatous polyposis were also not included as they were likely to be undergoing regular CRC surveillance due to their increased genetic risk. Potential participants were identified by practice receptionists and approached by the researcher prior to or following routine GP consultations, unrelated to a screening invitation.

### 2.2 Topic guide

The interview topic guide was based on the TDF.^15^ To evaluate the effectiveness, relevance and responses to the questions in the topic guide, three pilot interviews were undertaken with representatives of the target population. The final version of the topic guide is included as supplementary information. Although questions appeared to reflect a more structured interview, a flexible approach was adopted if participants wanted to discuss issues not in the topic guide.

### 2.3 Interviews

Face‐to‐face interviews were conducted in a private room in GP practices and began by ascertaining whether participants had received or completed a gFOBt. Participants were shown a sample gFOBt kit and instruction leaflet if they were not aware of screening. All interviews were audio‐recorded and transcribed verbatim. Participants also self‐completed a socio‐demographics questionnaire including questions on educational qualification, housing and vehicle ownership which were used to derive an individual index of SES^20^ which has shown greater associations with psychological characteristics than neighbourhood measures such as the Indices of Multiple Deprivation.^21^ Individuals who had an education qualification, owned their homes, including having a mortgage, and owned a vehicle were considered of high SES; those who owned a home or vehicle and had an education qualification were considered to be of intermediate SES; and individuals who did not own their home or a vehicle nor had any educational qualifications were considered to be of low SES.

### 2.4 Data analysis

Interviews were analysed using Framework Analysis^22^ as this approach was best suited to the a priori use of a theoretical framework in this study with constructs being determined by variables in the TDF. Transcripts were managed using Nvivo (QSR International Pty Ltd; Doncaster, Vic., Australia). The five steps of the Framework approach were followed including familiarization, developing a thematic framework, indexing, charting and interpretation.^22^ The coding and charting were done by one researcher (ND) and cross‐checked by a second researcher (AJW) to ensure there was consensus on the thematic content of the categories and how they fitted into the TDF domains. The TDF was used to initially code and categorize the data using a top‐down approach, and as analysis progressed, themes were also allowed to emerge bottom‐up and incorporated into the framework. Charts were created for each of the TDF domains and overarching themes and concepts were identified and examined for any patterns according to the ethnicity and SES.

## 3 Results

### 3.1 Participants

Fifty people (21 women and 29 men) were interviewed, reaching data saturation. Reasons for non‐participation often included lack of interest or time. One participant agreed to be interviewed, however, later withdrew as they did not want to sign the consent form. Three further participants withdrew following consent, two due to deteriorating health and one participant was going on holiday and a convenient time for interview could not be arranged. Participants' demographic details are summarized in Table 1. An individual‐level SES score for each participant was also assigned from the socio‐demographic information they gave.^20^ The majority of participants were within the intermediate SES category as they either had an educational qualification or owned their home or vehicle. There were noteworthy differences in SES between members of different ethnic groups, whereby the majority of African participants were in the intermediate category due to an educational qualification to at least tertiary level but lacking home ownership. In contrast, the high and low SES categories comprised a more even mix of Caribbean and White British participants. Participants from each of the ethnic groups were present across all SES categories, allowing the exploration of the patterning of people's beliefs about CRC screening according to both factors.

**Table 1:** Summary of socio‐demographic details of interviewed participants

	N (%)
Gender	
Men (mean age 65.61 years, SD 4.73)	29 (58)
Women (mean age 65.13 years, SD 4.54)	21 (42)
Ethnicity	
Black African	13 (26)
Black Caribbean	15 (30)
Black Other	2 (4)
White British	16 (32)
White Other	4 (8)
Socio‐Economic Status (SES)^a^	
Low SES	17 (34)
Intermediate SES	22 (44)
High SES	10 (20)
Missing	1
Self‐reported screening status	
Completed	18 (36)
Declined	7 (14)
Not invited	19 (38)
Invited but not yet completed	5 (10)
Completed GFOBt as part of medical investigation	1 (2)
Marital status	
Single	16 (32)
Married/civil partnership or cohabiting	19 (38)
Separated or divorced	9 (18)
Widowed	6 (12)
Level of education^a^	
No formal qualifications	19 (38)
High school education ≤16 years	3 (6)
High school/college education ≤18 years	7 (14)
University education >18 years	11 (22)
Other (e.g. nursing qualifications, trade certificate)	9 (18)
Missing	1
Occupational status^a^	
Full‐time employment	5 (10)
Part‐time employment	6 (12)
Unemployed	3 (6)
Retired	30 (60)
Retired early	3 (6)
Other (e.g. voluntary work)	2 (4)
Missing	1
Housing tenure^a^	
Own home (including with a mortgage)	11 (22)
Renting	36 (72)
Other private accommodation	2 (4)
Missing	1
Car ownership^a^	
Yes	12 (24)
No	37 (74)
Missing	1
Time as UK resident	
From birth	17 (34)
<5 years	1 (2)
6–15 years	7 (14)
16–30 years	6 (12)
>31 years	19 (38)

a Denotes missing values where information was not provided.

The emergent themes related to the benefits of screening, prior awareness, fear of cancer, religious faith, civic duty and practicalities of gFOBt completion were derived from the broader domains of the TDF, as shown in Table 2.

**Table 2:** Barriers and facilitators to uptake of colorectal cancer (CRC) screening

Theme	Facilitators of uptake	Barriers to uptake	Domain of TDF
Benefits of screening	Helping oneself Helping others		Beliefs about consequences
Prior awareness	Previous experience of cancer and/or screening Raise awareness of screening	Lack of awareness	Knowledge
Fear of cancer		Fear of cancer/screening outcome	Emotions
Religious faith	God helps those who help themselves Religion and health are separate	Faith in God that one will not get CRC	Social role & identity
Civic duty	Civic duty		Social role & identity
Practicalities of completing the FOBt	Planning completion of the FOBt within daily schedule	Collecting faecal sample Physical/mobility limitations Misunderstanding of information/instructions Other priorities/lack of time	Behavioural regulation

#### 3.1.1 Benefits of screening

##### Helping oneself

A consistently reported facilitator of screening by participants of all ethnic and SES backgrounds was the belief that taking part in screening was a way of protecting one's own interests and keeping healthy, a particular priority as participants reported feeling more susceptible to illness as they were getting older. Whilst this finding may reflect a limitation of recruiting participants from general practices, many participants, regardless of health status, believed cancer was a hidden disease that developed silently inside the body. Furthermore, many participants believed that if cancer was present, early detection via screening would result in more successful treatment and fewer complications and ultimately save one's life.

Participants of all ethnic and SES backgrounds also perceived screening as an opportunity to gain reassurance that they did not have CRC, particularly if they did not visit their GPs often.

…I was very interested, I find it very interested and I was very glad when I sent mine, because there was some doubt within me own self because the way I usually feel sometimes, and I don't visit my doctor very often and so forth, so I didn't sure whether I was…

(P52, Caribbean, male, 72 years, completed screening, low SES)

Repetition of screening every two years provided further reassurance to participants as they knew they would be monitored to ensure there had been no changes. However, White British participants of high SES tended to express greater doubts about the general effectiveness of screening, chance of false‐positive results and whether screening was entirely beneficial to individuals.

So a little bit of scepticism about screening, I was by and large thinking if it's done it will be worked out that on balance it's worth doing and worth the spending the money on. And some screenings not actually showing the condition as well, it showed something else, factors which may lead to the condition. There's various things in my mind that are not clearly logged, but some doubts about screening.

(P5, White British, male, 60 years, not completed, high SES)

##### Helping others

As well as helping oneself by taking part in screening, the majority of participants also believed that their participation would benefit others. This factor also encouraged participation in screening. The perception of helping others appeared to be intertwined with beliefs about the purpose of screening; whilst some participants believed screening was like having a regular check‐up, quite a few Black African and Black Caribbean participants in the intermediate and low SES groups thought it was a form of medical research. To that end, taking part in screening was perceived to have benefits for society in general as participation could advance science, and possibly help find a cure for CRC.

After I've done the screening, if you can use it to help other people, I think it, I think it would be great… I have a family coming up, my next generation and when I've done this screening, if one of them— hoping not—but if one of them might sick in that way, might be hope you can help them.

(P44, Caribbean, male, 71 years, not yet invited for screening, low SES)

An underlying motivation for many Black African participants across all SES categories was the need to complete screening for the sake of their families, be it to avoid distress for partners and children if they were later diagnosed or a desire to live and see their grandchildren grow up.

#### 3.1.2 Awareness of CRC

Many participants often knew a close family member or friend who had died of cancer and did not want to endure the same pain and suffering themselves as a reason for participating in screening. Some participants reported feeling susceptible due to a family history of cancer whilst for others it reinforced the perception that cancer was a nasty illness that could “creep up” on them at any time. Four participants had previously suffered with cancer themselves and thus believed they were at increased risk of getting CRC, so were keen to be screened. Thus, people's knowledge, by way of previous cancer experience and family history, appeared to influence their perceived susceptibility to CRC.

I had a very close friend who died of it, we were for many years close. So, and erm, I saw the whole process as such, I was with him throughout the period until he passed away… When you've seen someone close going through that process, then you understand why you have to fill in those, do those tests.

(P33, African, male, 60 years, completed screening, high SES)

Black African and Black Caribbean men tended to be more aware of prostate cancer but demonstrated very low awareness of CRC. Likewise, awareness of CRC was low in White British men who also were not familiar with the concept of screening.

Very little because I've not had to have any screening tests or anything like that. So it's very little, it's just a word.

(P23, White British male, 62 years, declined screening, intermediate SES)

The majority of participants reported they knew very little about CRC and only became aware of the screening programme on receipt of their invitation. As a result, the screening invitation came as a surprise to participants who were not previously aware of the screening programme. However, those who were previously aware of CRC screening accepted their invitation because it was expected.

I wasn't familiar with this one, so I don't know. It's more of the “don't know” factor. I didn't expect to get a cancer screening kit at sixty. I hadn't heard about it.

(P5, White British, male, 60 years, not completed, high SES)

Participants of high SES tended to question why screening was not more widely promoted in media campaigns or GP surgeries like initiatives such as flu immunization, as this was seen to encourage more people to take part in screening.

But the question is not done at the GP, it's not mentioned when they go to the GP, “Please do your tests, bowel cancer can catch‐up with anybody.”… So it could help by the GP's place, if the reception tells you, or even if the GP tells you, you could be a tremendous help, you know, to say—“Have you had this form? Please do it.” That's all, that's all they need […]It comes through the post, that's the end of it. It's good to just ask them or remind them to do it. You know, just like they remind everyone to take their flu jab.

(P33, African, male, 60 years, completed screening, high SES)

#### 3.1.3 Fear of cancer

Overall, fear of CRC and fear of the potential outcomes of screening such as a positive result tended to discourage participation in screening for White British participants of all SES backgrounds but not for participants from other ethnic groups. Whilst some participants feared a cancer diagnosis, others who were discouraged reported the stigma of cancer and feared ridicule if they discussed screening with others.

…I don't want to do it voluntarily. I suppose I'm scared of cancer…just one of those diseases that people with are shunned.

(P23, White British male, 62 years, declined screening, intermediate SES)

In contrast, many Black African and Black Caribbean participants of all SES backgrounds along with some high SES White British participants reported no fear or embarrassment of screening.

I just think, well it's nothing to this, there's nothing embarrassing or scary like that, with this, it's just a simple little thing, you just put it on there and cover it up and that's it.

(P11, Black Other, female, 69 years, completed screening, low SES)

#### 3.1.4 Religious faith

Religious faith overall encouraged screening participation for Black African and Black Caribbean participants holding either Christian or Muslim beliefs as screening was seen as a way of helping oneself. Moreover, those that thought they may get a positive result were not disheartened as they believed God would help them in case they had cancer.

God is above everything…the Bible says God help those who help themselves and by helping myself, is by coming to you to examine me to see if there is any problem and then if, the Master, God, will be able to assist.

(P39, African, male, 63 years, completed screening, intermediate SES)

I'm a Muslim, but I can do anything for my health…That doesn't affect religion, belief, or not. That seem like nonsense, because if you believe in something, why don't you believe in something to make you get well?

(P13, African, female, 68 years, invited but not yet completed, low SES)

Religious beliefs impacted the screening decision of one White British participant of low SES who reported not taking part due to the FOBt containing the term “occult” which had demonic connotations for her.

I don't want to be messing around with anything that's got anything to do with the occult…to me it brings up Satan and demonic things and, you know, and the bowels are very significant, you know, really, in the spiritual world

(P30, White British, female, 60 years, declined screening, low SES)

For two participants, one Black African and one Black Caribbean person, religious faith was linked to beliefs about perceived risk of CRC. As illustrated in the quotation below, one participant believed she would not get CRC with God's grace which discouraged her to participate in screening. However, by the end of the interview, the participant below (P34) had changed her viewpoint and reported she would participate in screening when she was invited as the misunderstandings she held about the procedure and amount of faecal sample required were now clear. This suggested that misunderstanding of the procedure of the FOBt rather than faith in God was the reason the participant was initially reluctant about undergoing screening.

I believe that by the grace of God, I will not go through such illness. So I believe nothing as such will happen to me, because I have God who is taking care of me. So I don't want the screening and all that, I don't bother.

(P34, African, female, 65 years, not yet invited, high SES)

…you tell yourself, whatever happens now, it's in God's hands… I think they wouldn't mind if they start seeing changes and that, and a test has to be done. But otherwise, I don't know if people would just—well that's how I feel, it wouldn't be everyone.

(P15, Caribbean, female, 74 years, not yet invited, intermediate SES)

#### 3.1.5 Civic duty

A repeated theme underpinning many Black African and Black Caribbean participants' positive views about screening, regardless of SES, was a sense of “civic duty” to take part in screening because not participating would be a waste of the NHS time and money. Closer examination of the data highlighted that several White British participants of high SES also shared a similar perceived responsibility to participate in screening. However, for Black African participants in particular, screening was perceived as a privilege or a “help” and something that was not available in their native countries. The NHS was particularly valued as it was a free service that did not discriminate between the rich and poor, unlike the complicated and expensive health insurance policies of their country of origin. Again this finding may reflect the views of those who regularly use NHS services such as general practice.

Those of us who have the privilege of being in this country, are lucky with the care and technology. Where I come from, Nigeria, you don't have these. People dying of one thing or the other…the state doesn't have any provision for them, so they die.

(P46, African, female, 64 years, completed screening, intermediate SES)

An underlying sense of obligation to take part in screening was apparent for many Black African and Black Caribbean participants of high, intermediate and low SES, with connotations of a duty to abide by the rules of the country they were now living in.

…I know within myself that if people want to help you in this country, they say “do this”, you must do it, that's why when they send this specimens, this thing to me to send my specimens, I did it orderly, I send it and I'm happy that they give me feedback that everything was good.

(P39, African, male, 63 years, completed screening, intermediate SES)

#### 3.1.6 Barriers to FOBt completion

##### Everyday pressures

For the participants who did not complete their gFOBt kit when they had been invited for screening (n=7 White British participants from a range of SES backgrounds), discouraging factors included existing physical or mental health problems, being too busy or stressed at work, as well as caring for an elderly parent.

Well I did get a screening test through the post some years ago, but my circumstances at the time, I didn't get round to dealing with it, because I was caring for my mother who had severe dementia. So my entire time was full of doing that, so I just left it to one side, and didn't bother with it.

(P24, White British, female, 63 years, declined screening, intermediate SES)

##### Faecal sample

Collection of the faecal sample and returning the gFOBt via post tended to be an obstacle to screening for some White British participants across varying SES categories. Participants were concerned about potential smell, lack of hygiene as well as the mess that completing the FOBt may entail.

And I think I have to say that, that the fact that it involved, you know, sending off faeces, for example, didn't help, because it's, I think that one would have to be quite sort of, you know, committed and interested to do that.

(P14, White British, male, 62 years, declined screening, high SES)

However, none of the Black African and Black Caribbean participants were deterred or disgusted by the procedure of the gFOBt. Instead, they viewed collection of the faecal sample as a natural behaviour and equated it with other medical tests or procedures such as taking daily insulin. One Black African participant of high SES further justified the naturalness of the gFOBt procedure by comparing it to traditional practices of burying faeces in the ground in the absence of toilet facilities.

##### Misunderstanding of instructions

For participants who had either contemplated or attempted the gFOBt, misunderstanding of the instructions for completion was an obstacle to screening participation, mainly for those of lower SES. However, those who had difficulties but were motivated to participate either consulted, or planned to consult, a health‐care professional for advice on how to complete the test.

You sent one to me, which I was going to do, because I didn't understand how to do it, I was trying to bring it to the nurse here, so that she know exactly what to do with it.

(P13, African, female, 68 years, invited but not yet completed, low SES)

##### Planning gFOBt completion

Participants' perceived ability to prepare for how they would complete their gFOBt appeared to facilitate the completion of screening across all ethnic and SES groups. Another aspect related to gFOBt completion of the gFOBt for all participants was the need to ensure it could be scheduled around participants' regular daily work or leisure activities; the majority preferred to complete the test in the morning.

No, I think it's best in the morning, not during the day, because during the day I go to luncheon clubs and things like that. And I think it's best to do it first thing in the morning. And then it don't break up the routine.

(P22, White British, male, 66 years, completed screening, low SES).

## 4 Discussion

This study identified the beliefs about CRC screening of an ethnically and socio‐economically diverse group of people from south‐east London. Many beliefs were shared across the different ethnic and SES groups, such as screening's benefits including reassurance that one did not have CRC, with few barriers being endorsed exclusively by ethnic minority groups. However, participants of low SES, regardless of ethnicity, reported encountering more difficulties surrounding the comprehension of instructions for gFOBt completion than those of higher SES. This is consistent with previous research reporting lower screening intentions in deprived community settings^23^ and people with lower literacy^24^ who were less likely to complete the gFOBt. These challenges may be partly due to self‐completed nature of the gFOBt which, unlike other screening tests, requires invitees to adequately understand and independently act on complicated instructions for completion.

Previous studies have reported low knowledge of CRC screening in ethnic minority groups and people of low SES.^8^, ^11^, ^25^, ^26^, ^27^, ^28^ The present study found the same, but also showed knowledge was low in White British participants irrespective of SES. This finding emphasizes the importance of examining the views of both majority and minority ethnic group populations alongside socio‐economic factors to avoid misattributing belief patterns amongst members of minority ethnic groups to being due to their belonging to a minority ethnic group rather than being shared with others of similar SES. Furthermore, ethnic minority participants had greater awareness of CRC screening than their White British counterparts in an omnibus survey.^29^ In our interviews, we found that despite their low awareness of screening, the majority of participants knew someone with cancer. Black African and Black Caribbean participants in particular, regardless of SES, were more aware about prostate cancer, which has a higher incidence in these groups.^30^ A consistent message from participants in ethnic minority groups was the need to inform and promote screening through media campaigns and GP practices.

There were also some further notable differences between ethnic groups. Black African and Black Caribbean participants of all SES levels were more positive and accepting of screening than White British participants. They also did not perceive any fear of screening or report disgust at collecting the faecal sample unlike White British participants in our study and other minority groups in previous studies.^28^, ^31^ Furthermore, ethnic minority group participants reported a perceived civic duty to participate in screening as well as a need to conform to the rules of the country they now lived in. This is consistent with a previous study with White British participants.^12^ On the whole, faith in God encouraged screening participation for Black African and Black Caribbean participants from varying religious backgrounds, a finding that is unique to the present study.

### 4.1 Strengths and limitations

Although the CRC screening programme has significantly evolved since this study was conducted, our findings have provided an important contribution to understanding screening participation. The comprehensive theoretical framework allowed the identification of factors that are not usually included in theories of health behaviour, such as religious faith, participants' perceived civic duty to participate in screening, and practical aspects of completing the gFOBt. Furthermore, our study was unique in exploring how the personal beliefs of individuals map onto individual factors of ethnicity and SES, whilst unravelling the views of African and Caribbean groups separately in recognition of the distinctiveness of both groups. Our SES measure, comprising level of education, housing tenure and car ownership^20^ allowed a more holistic understanding of the impact of SES on beliefs about CRC screening. Previous UK studies^11^, ^31^ examining socio‐economic variation in CRC screening via gFOBt have relied on the index of multiple deprivation (IMD,^32^). As IMD is an area‐based measure of deprivation, there will be individuals whose personal social circumstances are not well described by their area of residence's IMD score.

Some potential limitations of this study also warrant attention. Firstly, this study focused on a sample of participants who were recruited in GP practice waiting rooms. Although the GP setting allowed wide access to the local populations of Lambeth and Southwark, this may have led to biases in sampling by accruing participants who more motivated about their health and engaged with health services. It may be that participants who were already “patients”, such as older adults, may have had different attitudes, motivations and beliefs about screening than those who do not visit their GPs often. However, similar findings have been reported in studies sampling from community settings.^31^ A further sampling bias may be that as many Black African and Black Caribbean participants were highly educated, with some previously having worked as nurses, which may have facilitated their understanding of screening. Due to different migration history, cultural patterns and environmental conditions, caution must also be taken when extrapolating these findings to other ethnic minority populations as this study only focused on members of Black African and Black Caribbean groups.

### 4.2 Implications for practice

The findings of this study have some important implications for improving access to CRC screening via gFOBt. Our findings showed that people were lacking procedural knowledge on how to complete the gFOBt. Recent studies trialling the impact of increased knowledge of the purpose and possible outcomes of screening did not demonstrate any impact on screening uptake.^33^ Very few studies^34^ have focused on the impact of increasing procedural knowledge on screening uptake so this is one potential research gap to explore.

Participants in our study wanted information about completing the gFOBt in more simplified, accessible and easy to understand formats. They also wanted more active promotion of screening by their GP practices and the NHS. A RCT in the UK showed that receiving a CRC screening invitation letter signed by participants' GP was associated with increased screening participation.^34^ This finding was replicated in a recent four‐cluster RCT in the UK where a GP endorsed reminder letter led to a reduction in the SES gradient in screening uptake.^33^The lack of screening promotion campaigns at the time our study was completed suggested to participants that screening was not important. These results echo previous findings of widespread preference amongst participants for a recommendation from the NHS to take part in CRC screening.^35^ However, alongside a preference for screening recommendations, participants in that study also expressed a strong desire for detailed information regarding screening's risks and benefits. Therefore, there is a clear imperative for practitioners to continue to provide risk–benefit information. Being able to plan completion of the gFOBt amongst work and daily activities also appeared to facilitate participation in screening. With the age range of the screening programme expanding shortly, it is important to have screening technologies that are both convenient and adaptable to people's lifestyles. Marked increases in screening uptake across population samples in the UK have been found in pilots of the faecal immunochemical test (FIT), a test that requires one faecal sample compared with three samples that are required for the gFOBt.^36^ In relation to attitudes, a recent Scottish study reported differences in perceptions of FIT and gFOBt where the FIT was perceived as less disgusting and easier to complete.^37^ Furthermore, SES predicted intentions to complete the gFOBt, but not the FIT suggesting the introduction of the FIT may be helpful in reducing SES disparities in screening uptake due to greater perceived disgust of the gFOBt.

## 5 Conclusion

Our findings suggest that there were considerable commonalities in the views of a diverse range of south London residents about some aspects of CRC screening, particularly awareness and the perceived benefits of screening. However, lower SES individuals wanted greater explanation of how to perform the test. Furthermore, our findings also demonstrate that perceptions of CRC screening are shaped by a multitude of psychological factors and lived experiences, which vary greatly between individuals. Quantitative studies examining the impact of this broad range of factors on screening uptake in different groups are required. There is a need to ensure that the CRC screening programme is a fair and equitable service where all invitees have an equal opportunity of making an informed choice.

## Funding

This research was funded by a doctoral studentship awarded to Nimarta Dharni by the Department of Primary Care & Public Health Sciences, Kings College London. We also thank the King's College Hospital Charity for funding part of the research.

## Conflicts of Interest

None.
