# Supplementary material for: Factors influencing participation in colorectal cancer screening—a qualitative study in an ethnic and socio‐economically diverse inner city population
Source: Health Expect. 2016 Aug 22;20(4):608–17. doi: 10.1111/hex.12489 (PMC5513014; doi:10.1111/hex.12489)
Supplement: Supplementary file 1 [file HEX-20-608-s001.docx]

**Supplementary table 1: TDF domains and interview questions**

| **Domain** | **Constructs** | **Question** |
| --- | --- | --- |
| Knowledge | Knowledge about CRC and screening  Procedural knowledge | Have you previously heard of bowel cancer, also known as colon, rectal or colorectal cancer?  What do you understand about bowel cancer? What do you think it is?  What is your understanding of screening?  Have you previously heard about the home screening test for bowel cancer...or done it yourself/ know anyone who has completed it? |
| Nature of the behaviour | What is the behaviour | What are your thoughts about you doing this test? |
| Behavioural regulation | Barriers and facilitators  Action planning | What factors may encourage/help you to complete the test?  What, if anything, would need to change in order for you to do the test? |
| Beliefs about capabilities | Self-efficacy  Control | How confident do you feel about completing the FOB test yourself?  Can you think of any difficulties you might have in completing the test? |
| Beliefs about consequences | Outcome expectancies | What do you think would be the benefits of completing the FOB test?  What would be the disadvantages or negative aspects of completing the FOB test? |
| Emotion | Affect  Fear  Anticipated regret | Looking at the test, I wonder what emotions you might feel when this comes through the post? (or for completers: thinking back to when the test came through the post, can you remember the emotions you felt?)  And what emotions do you think you might feel when completing the test itself?  How do you think you might you feel after doing the test? |
| Environmental context and resources | Resources  Environmental stressors | Is there anything you can think of in your everyday routine that may prevent (and help) you doing the test?  If you decide to do the test, would there be any factors preventing you from completing it? |
| Memory, attention and decision processes | Memory  Attention  Decision making | What kinds of things would you think about when deciding to do the test? (or when you were deciding whether to take part in screening, what factors came to mind?) |
| Motivation and goals | Intention  Goal priority  Intrinsic motivation | How important is to you to do this test for yourself?  How important do you think it is that this type of screening was introduced?  How likely is it that you will complete the FOB test when the invitation arrives (again)? |
| Skills | Skills  Competence  Ability | Looking at the test kit and the information alongside it, what skills do you think you might need to complete the FOB test? |
| Social influences | Social support  Social norms | What might people close to you e.g. partner, children, and friends, think about you doing the FOB test? |
| Social role and identity | Identity | Some people have religious or cultural beliefs that might affect whether they take part in (bowel cancer) screening. What are your thoughts about this? |
